# Locations of membrane protein production in a cyanobacterium

**DOI:** 10.1128/jb.00209-23

**Published:** 2023-10-03

**Authors:** Moontaha Mahbub, Conrad W. Mullineaux

**Affiliations:** 1 School of Biological and Behavioural Sciences, Queen Mary University of London, London, United Kingdom; 2 Department of Botany, Jagannath University, Dhaka, Bangladesh; Philipps-Universitat Marburg Fachbereich Biologie, Marburg, Germany

**Keywords:** cyanobacteria, cytoplasmic membrane, membrane proteins, mRNA, ribosome, thylakoid membrane

## Abstract

Cyanobacteria show an unusually complex prokaryotic cell structure including a distinct intracytoplasmic membrane system, the thylakoid membranes that are the site of the photosynthetic light reactions. The thylakoid and plasma membranes have sharply distinct proteomes, but the mechanisms that target proteins to a specific membrane remain poorly understood. Here, we investigate the locations of translation of thylakoid and plasma membrane proteins in the model unicellular cyanobacterium *Synechococcus elongatus* PCC 7942. We use fluorescent *in situ* hybridization to probe the locations of mRNAs encoding membrane-integral proteins, plus Green Fluorescent Protein tagging of the RplL subunit to reveal the location of ribosomes under different conditions. We show that membrane-integral thylakoid and plasma membrane proteins are translated in different locations. Thylakoid membrane proteins are translated in patches at the innermost thylakoid membrane surface facing the nucleoid. However, different proteins are translated in different patches, even when they are subunits of the same multiprotein complex. This implies that translation is distributed over the proximal thylakoid surface, with newly inserted proteins migrating within the membrane prior to incorporation into complexes. mRNAs encoding plasma membrane proteins form patches at the plasma membrane. Ribosomes can be observed at similar locations near the thylakoid and plasma membranes, with more ribosomes near the plasma membrane when conditions force rapid production of plasma membrane proteins. There must be routes for ribosomes and mRNAs past the thylakoids to the plasma membrane. We infer a system to chaperone plasma membrane mRNAs to prevent their translation prior to arrival at the correct membrane.

**IMPORTANCE:**

Cyanobacteria have a complex and distinct membrane system within the cytoplasm, the thylakoid membranes that house the photosynthetic light reactions. The thylakoid and plasma membranes contain distinct sets of proteins, but the steps that target proteins to the two membranes remain unclear. Knowledge of the protein sorting rules will be crucial for the biotechnological re-engineering of cyanobacterial cells, and for understanding the evolutionary development of the thylakoids. Here, we probe the subcellular locations of the mRNAs that encode cyanobacterial membrane proteins and the ribosomes that translate them. We show that thylakoid and plasma membrane proteins are produced at different locations, providing the first direct evidence for a sorting mechanism that operates prior to protein translation.

## INTRODUCTION

Cyanobacteria are oxygen-evolving photosynthetic prokaryotes that exhibit highly differentiated membrane systems. In addition to the outer membrane and inner (plasma) membrane, most cyanobacteria contain an additional intracellular membrane system, the thylakoid membrane (1 – 3). The thylakoid membrane is the sole site of photosynthetic electron transport and the major site of respiration (2). The proteome of the thylakoid membrane is sharply distinct from that of the plasma membrane (1, 3). The thylakoid membrane is densely populated with photosynthetic and respiratory complexes and their associated assembly factors (1, 3). The plasma membrane houses an alternative respiratory pathway, but most of its proteome consists of transporters, sensors, and components of the motility apparatus (3).

As vital cellular processes are so sharply segregated between the cyanobacterial thylakoid and plasma membranes, it must be crucial to target proteins to the correct membrane. Protein targeting mechanisms in typical bacterial cells are well documented, but mechanisms of cyanobacterial protein targeting remain obscure, due in part to the lack of a successful *in vitro* protein translation assay and difficulty in isolating intact thylakoid membrane sacs from cyanobacteria (4). In bacterial cells, protein translocation can be post-translational or co-translational. For co-translational targeting, a leader sequence at the N-terminus of the nascent polypeptide chain is recognized by the signal recognition particle (SRP). The translating proteins along with the mRNA and the ribosome are then located at either the Sec (Secretory) or the YidC translocon, and the new polypeptide is inserted into the membrane *via* the translocon as it is translated (5). For post-translational targeting, the fully translated proteins may be translocated either in the unfolded state by the SecB-dependent Sec pathway or in the fully folded state by the Tat (Twin arginine translocase) pathway. In both cases, signal sequences in the polypeptide are required for targeting to the membrane (4, 6).

Translation-independent location of mRNA molecules encoding membrane-integral proteins at the plasma membrane has been observed in *Escherichia coli*, suggesting that signals in the mRNA molecules themselves can direct mRNAs to a membrane surface (7, 8). mRNA targeting is controlled by nucleotide content and secondary structure, and it can act as an alternative to the SRP-dependent pathway to ensure co-translational membrane insertion of membrane proteins (8). Translation-independent mRNA localization has also been reported in chloroplasts of a unicellular alga, *Chlamydomonas reinhardtii,* where the untranslated mRNAs encoding the D1 subunit located near the translation zone which is the site of Photosystem II (PSII) biogenesis (9).

Membrane proteins in cyanobacteria are translocated by the Sec and Tat pathways and are inserted into the membrane by the Sec-dependent SRP pathway (4). Sec and Tat translocons are found in both the thylakoid membrane and the plasma membrane (4). However, most cyanobacteria, including *Synechococcus elongatus*, contain just a single set of genes for each of these translocons, suggesting that the translocons in the thylakoid and plasma membranes must be similar (4, 10). Cyanobacterial membrane-targeted proteins generally contain N-terminal leader sequences specific for the Sec or Tat translocons, but no differences between the leader sequences for thylakoid and plasma membrane-targeted proteins have been detected (4). Accordingly, it has been suggested that both sets of proteins might be translated in membrane regions connecting the thylakoid and plasma membranes and then sorted post-translationally into one membrane or the other (1). However, cryo-electron tomography on cyanobacterial cells has failed to detect any direct connections between the lipid bilayers of the two membrane systems, although the membranes can run close to each other with protein bridges spanning the gap (11, 12).

We recently studied the subcellular localization of mRNAs encoding the thylakoid-located photosynthetic core proteins in two species of cyanobacteria, finding that these mRNAs are located in clusters at the inner surface of the thylakoid membrane system adjacent to the central cytoplasm (13). Thylakoid membrane affinity of the mRNAs is retained even in the absence of translation and ribosome association (13). This translation-independent localization of mRNAs suggests that the specific thylakoid targeting signal could reside in the mRNAs rather than in the proteins. We identified two RNA-binding proteins (RBPs) that are implicated in localizing the photosynthetic mRNAs at the thylakoid surface and we suggested that these RBPs might recognize photosynthetic mRNAs and chaperone them to the thylakoid surface (13). In the absence of the RBPs, mRNA localization is perturbed. The photosynthetic complexes are still assembled at the thylakoid membranes but the cells respond slower to changing light conditions, suggesting that the efficiency of photosystem biogenesis is impaired (13).

The mechanisms of membrane targeting and the locations of translation and membrane insertion of cyanobacterial plasma membrane proteins remain unclear. Here, we further investigate the sites of translation of cyanobacterial membrane proteins by probing thylakoid and plasma membrane mRNAs and by green fluorescent protein (GFP) tagging of ribosomes. We selected as our model organism the rod-shaped unicellular cyanobacterium *S. elongatus* PCC 7942 (hereafter, *Synechococcus*) because its smooth and regular thylakoid membrane organization is advantageous for quantitative fluorescence microscopy (12, 14, 15). We conclude that thylakoid protein translation takes place at multiple sites on the innermost thylakoid membrane surface while plasma membrane protein translation occurs at the plasma membrane. This indicates a pathway for ribosomes and mRNAs through the thylakoid system to the plasma membrane and a sorting mechanism that operates prior to translation.

## RESULTS

### Photosynthetic protein translation zones

**Fig 1:**
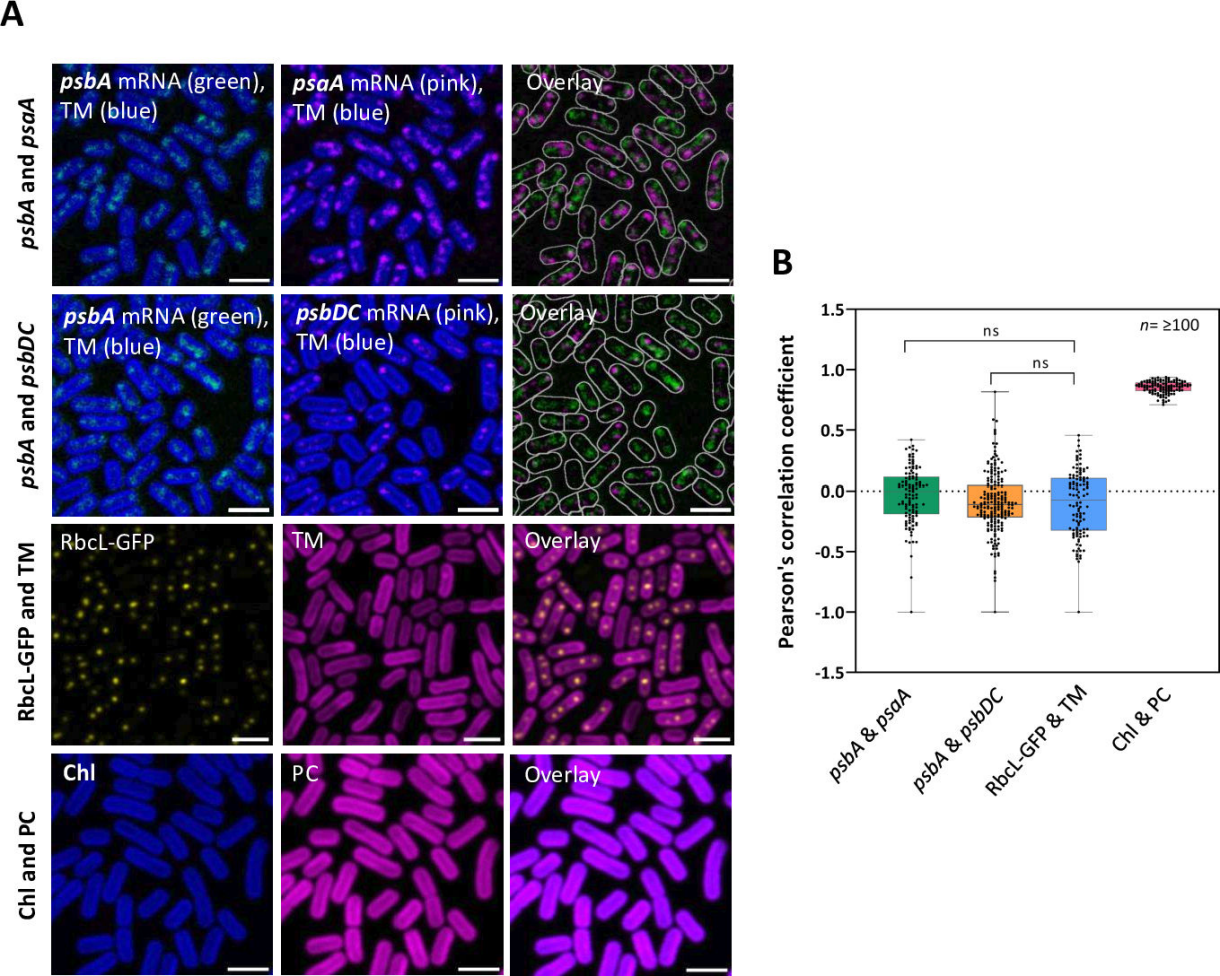
Colocalization analysis of pairs of mRNA species encoding photosynthetic protein subunits. (**A**) Fluorescence micrographs showing RNA-FISH signals for *psbA* and *psaA* (top row) and *psbA* and *psbDC* (second row), showing the FISH signals relative to photosynthetic pigment fluorescence from the thylakoid membranes (TM). The overlay images show both FISH signals but omit the TM fluorescence: the white borders indicate the cellular outline. The third and fourth rows show pairs of fluorescence signals used as control standards for the colocalization analysis: RbcL-GFP (13) vs TM; chlorophyll (Chl) vs phycocyanin (PC). All scale bars are 2 µm. (**B**) Calculation of Pearson’s correlation coefficient for each of the pairs of fluorescence signals shown in (**A**). Error bars in the box plots indicate the range of values recorded, the center line shows the median, and the box spans the interquartile range, *n*: the number of cells measured, ns = *P*-value non-significant, measured by unpaired two-tailed Student’s *t*-test.

We previously probed the locations of several mRNAs encoding core subunits of the photosynthetic reaction centers in *Synechococcus* and found each mRNA clustered in comparable spots near the innermost thylakoid membrane surface (13). Although thylakoid association is independent of translation, the clustering of the mRNAs into tight foci does appear to depend on active translation, suggesting that the foci represent zones for translation and membrane insertion (13). To test whether the foci represent common translation zones for all reaction center components, we simultaneously probed pairs of mRNA species. The spectral window for fluorescent *in situ* hybridization (FISH) detection in cyanobacteria is strongly constrained by the highly fluorescent photosynthetic pigments, but TAMRA (5-carboxytetramethylrhodamine), with peak fluorescence at 578 nm, and FAM (fluorescein), with peak fluorescence at 520 nm, provide labels that can be readily distinguished from each other and the photosynthetic pigments. For the first mRNA pair, we used a FAM-labeled probe-set for *psbA*, encoding the D1 subunit of PSII, combined with a TAMRA-labeled probe-set for *psaA* mRNA, encoding a PSI core subunit (Fig. 1A). The *psbA* probe-set was designed against the highly expressed *psbA1* gene (16), but the nucleotide sequence conservation with the other *Synechococcus psbA* genes means that FISH experiments will not distinguish between members of the *psbA* gene family (13). For the second mRNA pair, we combined the FAM-labeled *psbA* probes with TAMRA-labeled probes for the *psbDC* locus encoding the D2 and CP43 subunits of PSII (Fig. 1A). All three mRNA species appear in discrete foci; however, there is no obvious colocalization between the pairs of mRNAs probed (Fig. 1A). We quantified the extent of colocalization with Pearson’s correlation coefficient, which returns values of 1 for perfect correlation, −1 for perfect anti-correlation, and 0 for uncorrelated signals (17). As a negative control for correlation, we used a *Synechococcus* strain with RbcL (the large subunit of ribulose-1,5-bisphosphate carboxylase) tagged with GFP (18, 19). RbcL is a cytoplasmic protein mainly packaged into carboxysomes, and accordingly, RbcL-GFP appears in foci in the central cytoplasm (18, 19). In our measurements, RbcL-GFP fluorescence showed slight anticorrelation with chlorophyll fluorescence from the thylakoid membranes (Fig. 1B): presumably, the tight spacing across the short axis of the cells prevents stronger anticorrelation in images obtained at optical resolution. As a positive control for correlation, we used chlorophyll and phycocyanin fluorescence. Both pigments are thylakoid membrane associated and we obtained correlation values close to 1 (Fig. 1B). Correlation values for the two pairs of mRNA probes were close to zero, suggesting random location relative to each other or slight anticorrelation (Fig. 1B). Thus, our results suggest that distinct photosynthetic mRNA species are each clustered and translated in their own separate zones at the proximal thylakoid surface.

### Location of an mRNA encoding plasma membrane integral proteins

Because of high background autofluorescence across the spectrum in cyanobacteria, mRNA-FISH can reliably detect only abundant mRNA species (13). Under steady-state conditions, the abundance of most cyanobacterial plasma membrane transcripts appears rather low in comparison to the transcripts encoding photosynthetic core subunits, for example (20). However, some plasma membrane transcripts are sharply up-regulated in response to stress or changing conditions. An example is the *Synechococcus nirA-nrtABCD-narB* operon (21), which is strongly induced by a switch from a medium containing ammonia as the nitrogen source to a medium containing nitrate (22). This operon includes the *nrtABCD* gene cluster, encoding a nitrate-specific plasma membrane active transport system, as well as *nirA* and *narB* encoding, respectively, nitrite and nitrate reductases (23 – 25). In the nitrate transport system, NrtB is the plasma membrane integral permease, NrtC and NrtD are the transmembrane ATPases, and NrtA is the nitrate receptor which is anchored to the plasma membrane by a flexible linker (25, 26). To examine the location of this transcript, we designed a set of FISH probes mostly against the *nrtB* mRNA sequence within the operon (Table S2).

**Fig 2:**
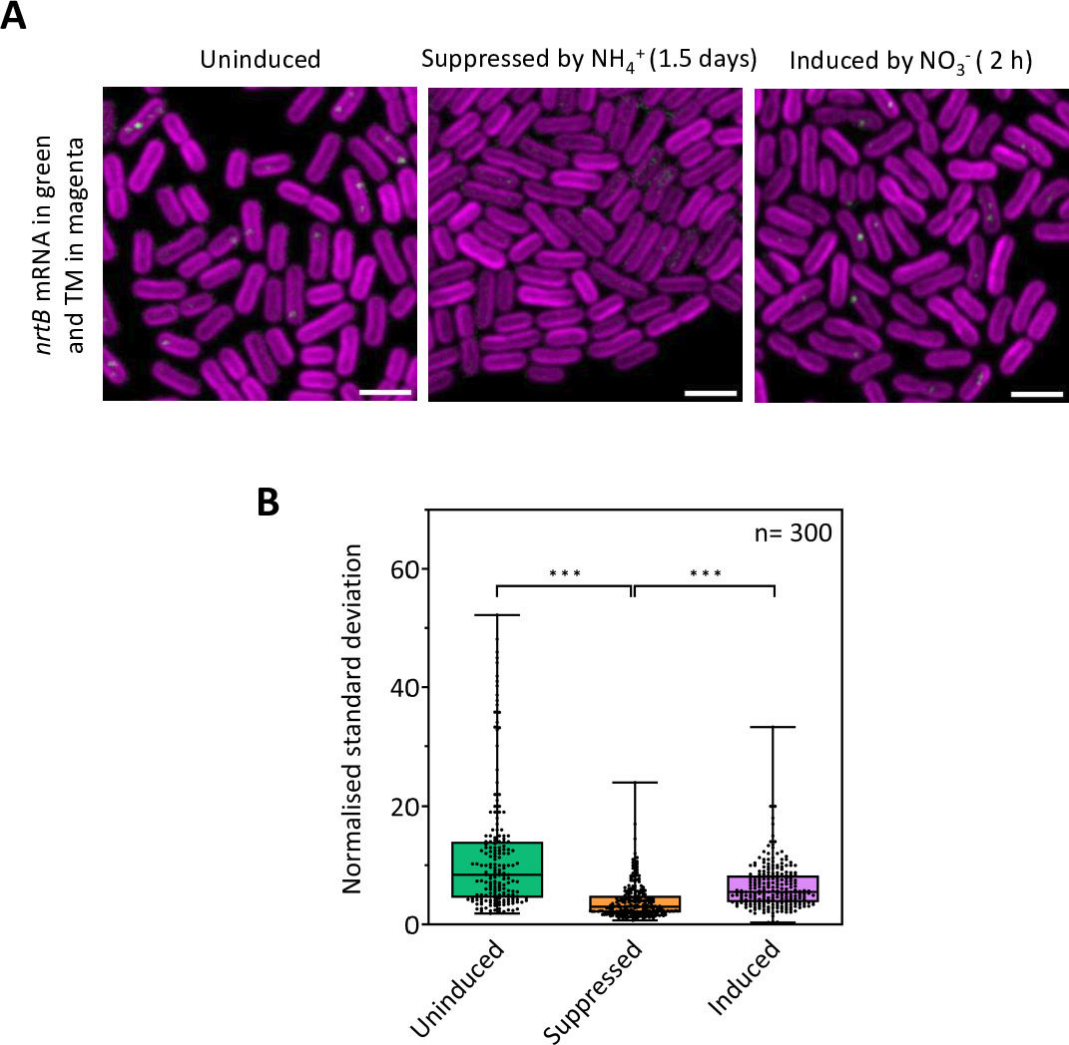
*nrtB* mRNA expression in cells grown in different conditions. (**A**) Fluorescence micrographs showing *nrtB* mRNA FISH signals in green and the thylakoid membrane (TM) in magenta. Scale bars: 2 µm. Uninduced cells were grown in a standard BG11 medium. Suppressed cells were grown for 1.5 days in basal medium lacking NO_3_^−^ but supplemented with NH_4_^+^. Induced cells were transferred from NH_4_^+^ to NO_3_^−^ medium and then fixed for FISH probing after 2 h. (**B**) Levels per cell of the *nrtB* mRNA signal are assessed by the normalized standard deviation in the FISH channel, which highlights the punctate FISH signals rather than the diffuse autofluorescent background. *P* values: 9 × 10^−19^ for untreated vs suppressed; 1.6 × 10^−14^ for suppressed versus induced. Error bars in the box plots indicate the range of values recorded, the center line shows the median, and the box spans the interquartile range. *n* = number of cells measured; *** indicates a significant difference (*P* < 0.001), measured by unpaired two-tailed Student’s *t*-tests.

FISH with the *nrtB* probes shows the expected regulation of *nirA-nrtABCD-narB* expression (Fig. 2). In cells grown in ammonia-containing medium, *nrtB* mRNA is almost undetectable (Fig. 2A), as expected from the strong repression previously observed under these conditions (22). A switch to an ammonia-free nitrate-containing medium is expected to rapidly induce *nirA-nrtABCD-narB* transcription (22). Within 2 h of making this switch, we observed the appearance of sharp foci that must correspond to *nrtB* FISH signals (Fig. 2A). Comparable FISH foci were observed in cells grown continuously in standard BG11 medium (Fig. 2A). Although bright, the *nrtB* FISH signals were localized and sporadic. Their total fluorescence was very weak in comparison to the diffuse autofluorescent background from the cells, making it problematic to quantify the total mRNA level in the cells. Therefore, rather than quantifying the total fluorescence signal per cell, we quantified instead the standard deviation in the signal, which highlights the sharp foci of the FISH signals. By this metric, there is clear suppression of *nrtB* expression in ammonia-containing medium and induction within 2 h of transfer to nitrate-containing medium (Fig. 2B). From our data alone, we could not be sure whether the appearance of the FISH foci upon transfer to nitrate-containing medium reflects new mRNA synthesis or the redistribution of existing mRNA. However, dot-blot analysis showed strong repression of the transcript in an ammonia-containing medium (22), so the appearance of the FISH foci almost certainly results from new mRNA synthesis.

**Fig 3:**
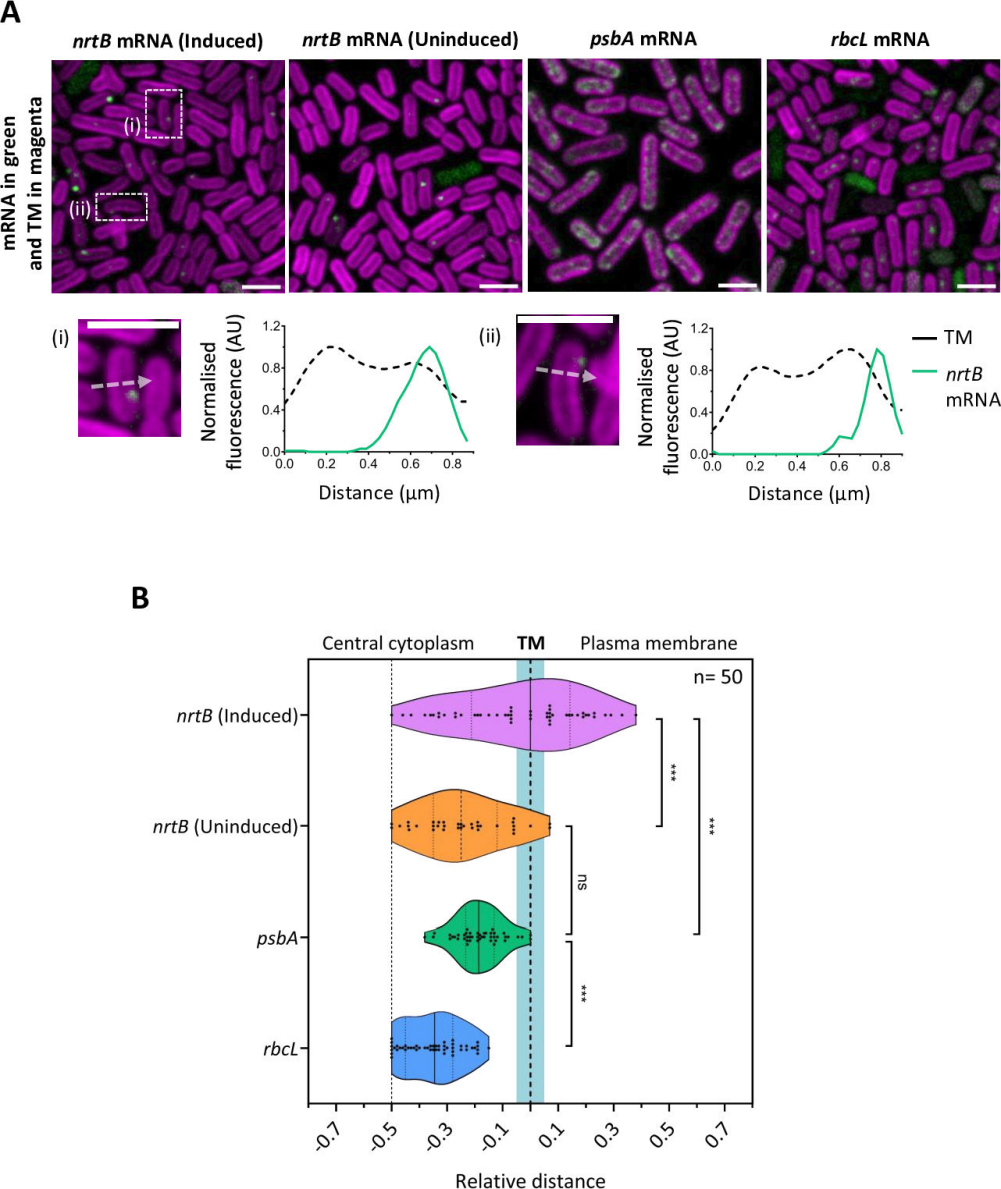
Location of *nrtB* mRNA relative to the thylakoid membrane. (**A**) Fluorescence micrographs showing FISH signals from different mRNAs (in green) relative to thylakoid membrane autofluorescence (TM, magenta). *psbA* and *rbcL* are shown for comparison as examples of mRNAs-encoding, respectively, thylakoid membrane and cytoplasmic proteins. Representative cell images (A i, ii; with line profiles as indicated) are both for induced cells and highlight two examples of *nrtB* FISH signals that peak outside of the thylakoid membrane. Scale bars: 2 µm. (**B**) Violin plots showing the radial distance of FISH foci from the center of the cell, relative to the thylakoid membranes (TM). The broken line indicates the peak of the TM fluorescence signal, with the approximate extent of the thylakoid zone shown in blue. Distances are shown along the short axis of the cell and are relative to the distance between the 2 TM peaks. *** indicates a significant difference (*P* < 0.001) and ns indicates no significant difference, as measured by unpaired two-tailed Student’s *t*-tests. *P-*values are 1 × 10^−5^ for *nrtB* induced vs uninduced; 6.8 × 10^−5^ for *nrtB* induced vs *psbA;* 0.078 for *nrtB* uninduced vs *psbA;* 4.6 × 10^−11^ for *psbA* vs *rbcL*. *n* = number of cells measured for each condition.

We observed some foci of *nrtB* mRNA in the central cytoplasm, but we also observed foci overlapping the thylakoid region and outside the thylakoids, where they must be adjacent to the plasma membrane (Fig. 3A). Two examples of this location in induced cells are highlighted in Fig. 3A (i) and (ii). By contrast, we previously showed that mRNAs-encoding cytoplasmic and thylakoid membrane proteins are located in foci in the central cytoplasm or at the innermost thylakoid surface (13). We never observed these species at the plasma membrane and rarely observed them spanning the thylakoid membrane region (13). Figure 3B quantifies the subcellular distributions of *nrtB* mRNA relative to the thylakoid membrane, in comparison to *psbA* and *rbcL* mRNAs encoding, respectively, a thylakoid membrane protein and a cytoplasmic protein. As previously shown (13), *psbA* and *rbcL* mRNAs are confined to the central cytoplasm, with *psbA* mRNA significantly closer to the proximal surface of the thylakoid system (Fig. 3B). In uninduced cells grown continuously on a standard medium, *nrtB* mRNA is also mainly found in the central cytoplasm, with very occasional foci at or beyond the thylakoids (Fig. 3B). However, in cells in which *nrtB* expression was freshly induced by transfer from ammonia-containing medium to nitrate-containing medium, *nrtB* mRNA distribution was significantly different, with around 50% of FISH foci overlapping the thylakoids or outside the thylakoid zone and therefore in the vicinity of the plasma membrane (Fig. 3B). This suggests that high levels of NrtB protein production correlate with the presence of *nrtB* mRNA at the plasma membrane.

### Subcellular distribution of ribosomes

**Fig 4:**
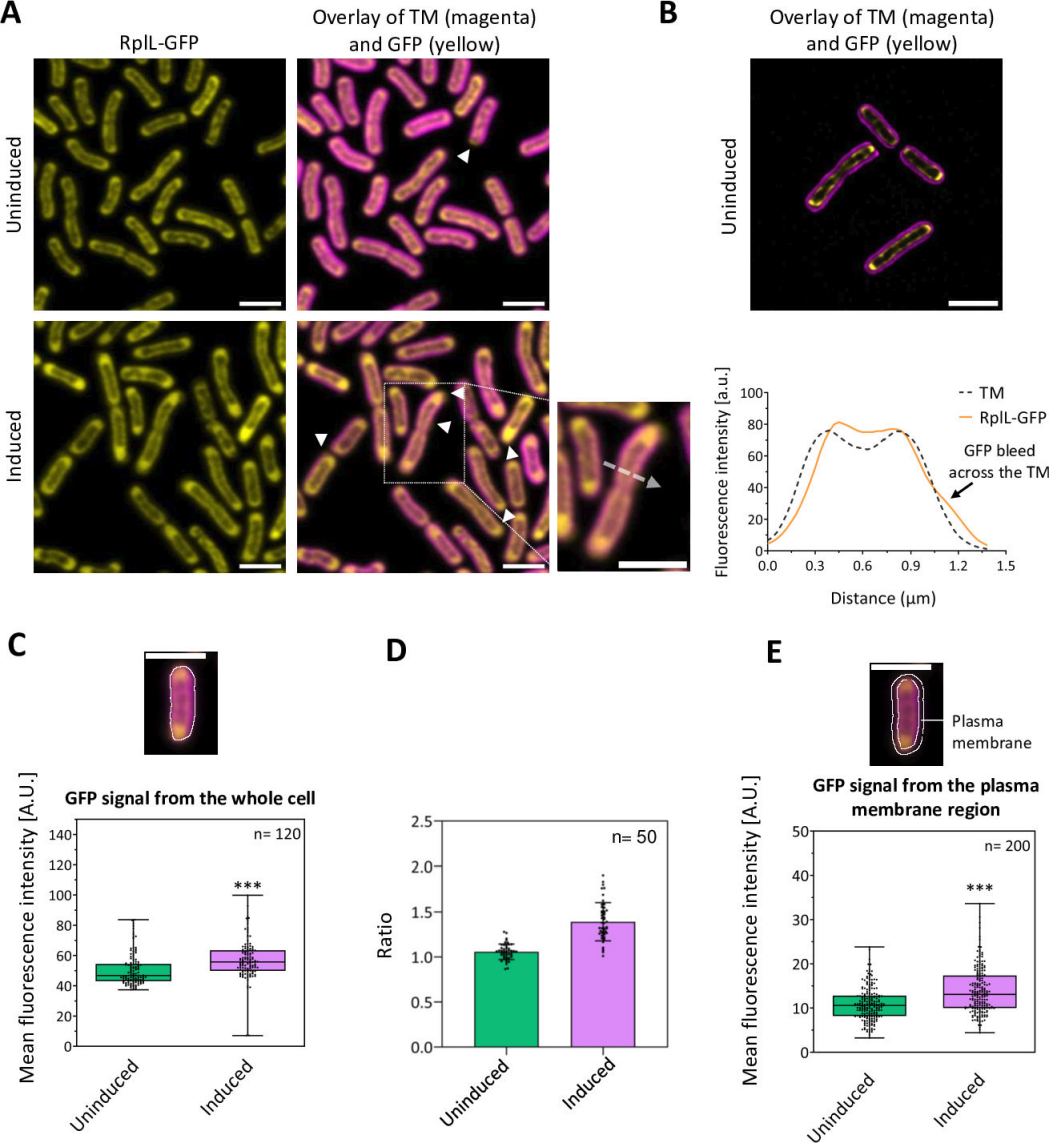
Subcellular locations of GFP-tagged 50S ribosomal subunits (RplL-GFP) in *Synechococcus* cells in different conditions. (**A**) Fluorescence micrographs showing RplL-GFP (yellow) distributions in cells uninduced or induced for *nrtB* expression as in Fig. 2. Thylakoid membrane (TM) fluorescence is shown in magenta. Arrows highlight some examples of GFP fluorescence signals appearing outside the thylakoid membranes, also shown in the short axis line profile from a representative cell. (**B**) Super-resolution fluorescence image showing the typical patchy distribution of RplL-GFP along the inner surface of the thylakoid membrane. (**C**) RplL-GFP signal per cell (*P* = 1 × 10^−5^ comparing *nrtB-*induced and uninduced cells). (**D**) Polar:equatorial ratio of RplL-GFP signals from *nrtB*-induced and uninduced cells. (**E**) RplL-GFP signal intensity from the plasma membrane region of the cell (*P* = 1.1 × 10^−10^ comparing *nrtB*-induced and uninduced cells). Error bars in the box plots indicate the range of values recorded, the center line shows the median, and the box spans the interquartile range, *n*: the number of cells measured, ***= significant difference, at *P* < 0.001, measured by unpaired two-tailed Student’s *t*-test. Scale bars: 2 µm for all panels.

To further investigate locations of membrane protein production in *Synechococcus,* we GFP-tagged the ribosomes. We added an eGFP tag to the C-terminus of the RplL protein of the 50S ribosomal subunit (Fig. S1A), a location for fluorescent protein tagging previously used in *E. coli* and *Bacillus subtilis*, in which the tagging had no effect on growth rates (27, 28). Cyanobacteria are polyploid, so mutations causing loss of function of essential genes generate cell lines that retain a mixed population of chromosomes when maintained under antibiotic selection (29). Surprisingly, we were unable to obtain a completely segregated *Synechococcus rplL-gfp* mutant, even after 6-month growth with antibiotic selection (Fig. S1B). This implies that complete loss of the native RplL protein is lethal. Nevertheless, the PCR segregation test indicates that the vast majority of *rplL* loci must encode the eGFP-tagged protein since the mutant PCR product is more abundant than the wild-type product despite being substantially longer, which invariably results in less efficient PCR amplification (Fig. S1B). Western blotting with anti-GFP antibody showed that all detectable GFP in the cells was linked to RplL, and RplL-GFP was detectable in both the membrane and soluble fractions from the cells (Fig. S1C and S1D). Virtually all *rplL-gfp* cells showed strong GFP fluorescence signals (Fig. 4). The cells showed no major abnormalities in growth (Fig. S1E) or morphology (Fig. 4); however, chlorophyll fluorescence per cell was slightly lower in *rplL-gfp* compared to wild type grown in parallel (Fig. S2A) which suggests that the tagged ribosomes are less efficient for translation of photosynthetic complexes. In addition, *rplL-gfp* cells were slightly longer on average than the wild type (Fig. S2B). To check whether RplL-GFP was incorporated into translationally active ribosomes, we tested the effect of puromycin, which decouples ribosomes from mRNAs (30). Puromycin treatment leads to changes in the abundance and distribution of photosynthetic mRNAs in *Synechococcus* (13). We found that puromycin treatment leads to a significant shift in the distribution of RplL-GFP, quantifiable as a decrease in the patchiness of the GFP fluorescence (Fig. S2C through S2E). Puromycin would only be expected to induce changes in the distribution of ribosomes actively engaged in translation, so RplL-GFP must be incorporated into active ribosomes. Therefore, the distribution of RplL-GFP gives information on sites of translation.

In *rplL-gfp* cells grown under standard conditions, most showed the majority of the GFP lining the innermost surface of the thylakoid membrane system, as shown by confocal microscopy in Fig. 4A. Occasional cells showed higher levels of GFP fluorescence in patches in the central cytoplasm (Fig. 4A). Structured illumination microscopy (lattice SIM^2^, with resolution ~60 nm in the *xy* plane) confirms the typical somewhat patchy distribution of GFP fluorescence along the innermost thylakoid surface (Fig. 4B). Most cells showed higher concentrations of GFP fluorescence at the poles. The proportion of GFP fluorescence that might be plasma membrane associated was generally low, but some cells showed faint patches of GFP fluorescence extending beyond the thylakoid system into the plasma membrane region (Fig. 4A).

To see whether the population of plasma membrane-associated ribosomes might be higher in cells expressing the plasma membrane nitrate transporter, we compared cells switched from ammonia-containing medium to nitrate-containing medium as in Fig. 2 and 3. This condition resulted in a small but significant increase in the mean RplL-GFP signal intensity (Fig. 4C) and a higher proportion of ribosomes at the cell poles (Fig. 4D). We segmented cell images to isolate GFP fluorescence outside the thylakoid system that might originate from plasma membrane-associated ribosomes (Fig. 4E). The proportion of such fluorescence was always low, but it increased significantly upon *nrtB* induction (Fig. 4E), consistent with an increased population of plasma membrane-associated ribosomes available to translate plasma membrane-associated *nrtB* mRNA (Fig. 3). We confirmed that *nrtB* mRNA showed a shift in distribution toward the plasma membrane upon *nrtB* induction in *rplL-gfp* cells as well as in the wild type (Fig. S3).

## DISCUSSION

### Location of ribosomes in *Synechococcus* cells

Our results from GFP-tagging of the RplL protein of the 50S ribosomal subunit give a comprehensive picture of the subcellular distribution of ribosomes in a cyanobacterium, albeit with the proviso that the mutant is not fully segregated and therefore there must be at least a small population of unlabeled ribosomes in the cell. Some unincorporated RplL-GFP is also a possibility. In all cells, the majority of ribosomes are found in the central part of the cell inside the thylakoid membrane layers, usually with stronger concentrations at the cell poles (Fig. 4A and B). On average, about half the ribosomes are found at the poles (Fig. 4D). The polar population of ribosomes increases following a switch in growth medium (Fig. 4D): this may be a stress response since a similar shift of ribosomes to the poles was seen in *E. coli* under antibiotic stress (28). In most cells, a prominent population of ribosomes appears to form a patchy coating on the innermost surface of the thylakoid membrane system (Fig. 4A and B). In some cells, diffuse patches of ribosomes in the central cytoplasm are also prominent (Fig. 4A). Biochemical fractionation and western blotting indicate a significant membrane-bound population of ribosomes (Fig. S1C), consistent with the distribution observed by fluorescence microscopy. Treatment with puromycin, which decouples ribosomes from mRNA (30), led to a more diffuse distribution of RplL-GFP in the cell (Fig. S2C through S2E); however, fluorescence imaging suggests that a significant proportion of RplL-GFP remains membrane-associated (Fig. S2C). This implies that 50S subunits can be anchored to the innermost thylakoid surface even when not mRNA-associated. There is a precedent in *E. coli* for the puromycin-insensitive association of ribosomes with the cell membrane, presumably through direct coupling with the SRP receptor (31). We could detect a fraction of the RplL-GFP signal distal to the thylakoid membranes and therefore in the vicinity of the plasma membrane: this constituted on average about 23% of the total cellular GFP signal (Fig. 4C and E). Our results on ribosome distribution in *Synechococcus* are broadly in line with an analysis of ribosome distribution in the cyanobacterium *Synechocystis* sp PCC 6803 (hereafter *Synechocystis*) from cryo-electron tomography (11). *Synechocystis* has a different thylakoid membrane arrangement from *Synechococcus*, with spherical rather than rod-shaped cells. However, *Synechocystis* also shows ribosomes coating parts of the thylakoid membrane surfaces that are exposed to the central cytoplasm, with a smaller population associated with the plasma membrane (11).

### The nature of photosynthetic assembly zones

In *Synechocystis*, a complex of chlorophyll synthase and the high light-inducible protein HliD copurifies with the Photosystem II assembly factor Ycf39 and the YidC/Alb3 insertase, suggesting a highly coordinated center for translation, membrane insertion, and maturation of PSII subunits (32). More generally, membrane fractionation studies, mainly in *Synechocystis*, suggest the concentration of Photosystem II assembly factors in specific thylakoid domains (33). Our previous study of photosynthetic mRNA location in *Synechococcus* and *Synechocystis* revealed that mRNAs encoding several membrane-integral photosynthetic proteins are concentrated in patches at the innermost thylakoid surface facing the central cytoplasm (13). We suggested that these mRNA patches might correspond to photosynthetic assembly zones (13). Here, we selected *Synechococcus* as our model because the photosynthetic mRNA patches are rather dispersed along the innermost thylakoid membrane surface, allowing assessment of whether different mRNA species are found in the same patches by simultaneous two-color FISH probing. Surprisingly, we found no significant colocalization between the pairs of mRNAs that we tested: *psbA* and *psaA* (encoding, respectively, core components of PSII and PSI) and *psbA* and *psbDC* (encoding different PSII subunits) (Fig. 1). Furthermore, the thylakoid-associated ribosomes (Fig. 4) appear to occupy more of the thylakoid inner surface than any single mRNA species [(13) and Fig. 1]. This all suggests that the entire innermost surface of the thylakoid system constitutes an assembly zone for photosynthetic complexes. Photosystem subunits are not translated in the same locations unless they are encoded on the same mRNA molecule (as with PsaA/PsaB, PsbD/PsbC). Presumably, newly translated subunits must diffuse in the membrane to find their partners for complex assembly. The thylakoid surfaces near the poles of the cell are notably enriched in both ribosomes (Fig. 4) and photosynthetic mRNAs (13) but ribosomes and mRNAs are also plentiful at other parts of the membrane surface. The biogenesis of new reaction centers appears not to be closely coupled to the biogenesis of new thylakoid membrane since new membrane sacs seem to appear at the distal edge of the thylakoid system, adjacent to the plasma membrane (12).

The high background fluorescence in cyanobacteria has so far prevented us from quantifying the numbers of mRNA molecules present in FISH foci, as has been achieved in other bacteria (34). However, the variable brightness of FISH foci (Fig. 1 to 3) implies that most contain more than a single mRNA molecule. Self-association of like mRNA species could be a driver for the segregation of different mRNAs that we observe at the thylakoid (Fig. 1): there are precedents for homotypic mRNA clusters in eukaryotes (35).

### Protein targeting to the plasma membrane

There are several previous suggestions for the sites of translation of plasma membrane vs thylakoid membrane proteins in cyanobacteria. It was suggested that thylakoid membrane proteins are synthesized at the plasma membrane (36) and, later, that both thylakoid and plasma membrane proteins are synthesized at an intermediate membrane zone and sorted post-translationally (1). Given the density of ribosomes at the inner edge of the thylakoid system [(11), Fig. 4], and the proximity of this membrane surface to the nucleoid, we also considered the possibility that plasma membrane proteins might be translated at the thylakoid. In this scenario, the proximal thylakoid membrane surface would be analogous to the rough endoplasmic reticulum, the site of translation of plasma membrane proteins in eukaryotic cells. To resolve the question, we looked for the location of mRNA species encoding integral plasma membrane proteins. The *nirA-nrtABCD-narB* mRNA proved to be a rare example that was sufficiently abundant for detection by FISH. This operon encodes the NrtABCD plasma membrane nitrate import system and is strongly induced when cells are switched from an ammonium-containing medium to a nitrate-containing medium (21 – 25). We could detect the transcript from sporadic FISH signals (Fig. 2 and 3) that were sometimes located in the central cytoplasm, but in inducing conditions, about half were located distal to the thylakoid membranes and therefore in the vicinity of the plasma membrane (Fig. 3). Of the other mRNAs that we probed, encoding thylakoid membrane or cytoplasmic proteins, none could be detected at the plasma membrane (Fig. 3). Given that we also detect some ribosomes at the plasma membrane, with a slightly increased population under *nirA-nrtABCD-narB*-inducing conditions (Fig. 4), we conclude that the plasma membrane is the site of translation of the NrtABCD complex, and likely other plasma membrane proteins too.

**Fig 5:**
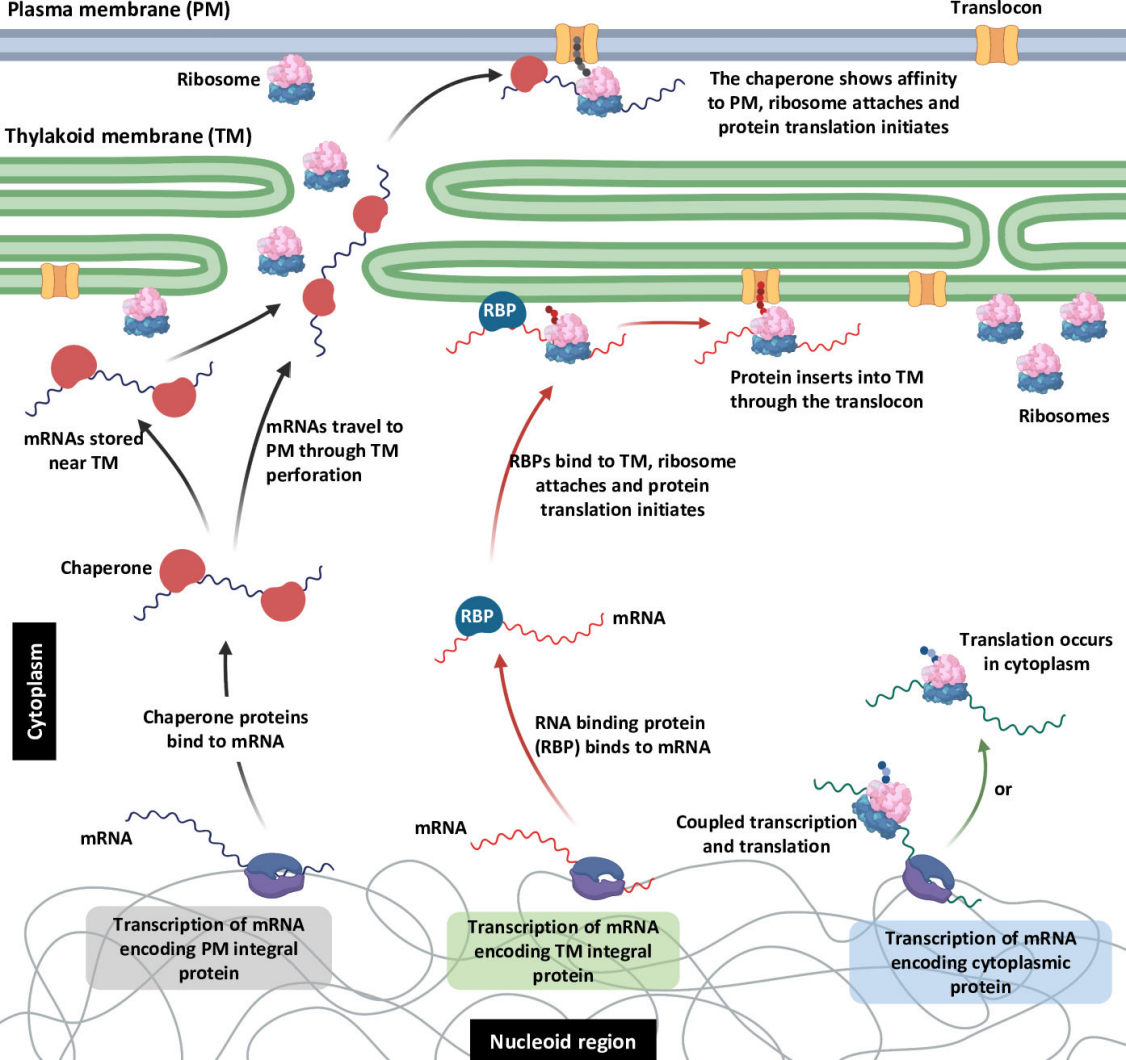
Model for protein targeting and membrane protein insertion in cyanobacteria. Plasma membrane proteins are translated and inserted at the plasma membrane, with an RNA chaperone that acts to prevent premature degradation of the mRNA and premature translation at the thylakoid and to promote the location of the mRNA at the plasma membrane. Thylakoid membrane proteins are translated and inserted at the proximal thylakoid membrane surface, with RNA-binding proteins promoting location at the thylakoid.

We previously showed in *Synechocystis* the involvement of RBPs in locating mRNAs-encoding thylakoid proteins at the thylakoid surface (13). A *Synechocystis* mutant lacking two RBPs showed a phenotype consistent with less efficient reaction center biogenesis, but nevertheless, reaction centers were still assembled and functional in the thylakoid membrane (13). It is likely that the thylakoid membrane is the default destination for membrane-integral proteins since the biogenic membrane surface faces the central cytoplasm and is close to the nucleoid (Fig. 5). Delivering mRNAs to the plasma membrane must be more challenging, requiring a route past multiple thylakoid layers to the plasma membrane and a chaperoning system to prevent the mRNA being captured and translated at the thylakoid. At low resolution, the cylindrical thylakoid layers of *Synechococcus* appear to form a continuous barrier between the central cytoplasm and the plasma membrane, but electron tomography revealed perforations in the thylakoid layers that are sometimes aligned to form a continuous passage from the central cytoplasm to the plasma membrane (37). Interestingly, these passages are often populated with ribosomes (37). Therefore, there clearly are routes for mRNAs and ribosomes to reach the plasma membrane. However, even in freshly induced cells, about half of the *nrtB* mRNA foci are located in the central cytoplasm (Fig. 3B). This would be consistent with the thylakoids as a rate-limiting barrier between the site of plasma membrane mRNA production in the central cytoplasm and its translation at the plasma membrane. This might necessitate storage of plasma membrane mRNAs for a substantial proportion of their lifetime in the central cytoplasm, whereas no such constraint would apply to thylakoid mRNAs, which are translated at the proximal thylakoid surface directly adjacent to the central cytoplasm (Fig. 5).

We hypothesize that there are one or more RNA-binding proteins that recognize specific features of plasma membrane mRNAs and chaperone them to prevent degradation or premature translation at the thylakoid membrane (Fig. 5); however, the existence and identity of these plasma membrane RBPs remain to be tested. Grad-seq analysis in *Synechocystis* revealed numerous new candidates for RNA chaperones (38).

## MATERIALS AND METHODS

### Strains and growth conditions

*Synechococcus elongatus* PCC 7942 cells were maintained in BG11 medium (39), supplemented with N-[Tris(hydroxymethyl)-methyl]−2-aminoethanesulfonic acid (TES) buffer (pH 8.2) at 30°C under constant white light (~20 µmol photons m^−2^s^−1^). Liquid cultures were grown in tissue culture flasks (Fisher Scientific) with continuous shaking (130 rpm) or maintained on BG11 plates containing 1.5% (wt/vol) Bacto-agar (VWR) supplemented with 0.3% (wt/vol) Na_2_S_2_O_3_. For the *rplL-gfp* strain, the medium was supplemented with 25 µg mL^−1^ chloramphenicol (Sigma-Aldrich).

### Construction of the *rplL-gfp* strain

The strain was generated in the *Synechococcus* wild-type background by inserting the *egfp* gene and chloramphenicol-resistant cassette (Cm^R^) downstream of the *rplL* (SynPCC7942_0631) gene (Fig. S1A). A linker (CTACCTGGTCCTGAACTACCT) was used between the *rplL* and *egfp* sequences. The NEBuilder HiFi DNA assembly master mix (NEB) was used to assemble a vector (pGEM-T Easy) carrying ~500 bp chromosomal sequence on either side of the insert to assist double homologous recombination. Primers used to amplify the DNA fragments prior to assembly are listed in Table S1. The assembled plasmid vector was cloned into NEB5-alpha competent *E. coli* cells (NEB). *Synechococcus* cells were transformed as in reference (40). Successful transformation and segregation status were checked by colony PCR (Fig. S1B).

### Puromycin treatment

Cells were treated with puromycin (500 µg mL^−1^) for 1 h under standard growth conditions and then collected by centrifugation (3,000 *g*, 10 min) and fixed with 1 mL PBS (phosphate-buffered saline, Life Technologies/Ambion) containing 3.7% (vol/vol) formaldehyde (Fisher Scientific) with incubation at room temperature for 30 min.

### Transcriptional regulation of the nitrate transporter

To suppress transcription, cells were washed and cultured for 1.5 days in a basal medium supplemented with 3.75 mM of (NH_4_)_2_SO_3_. In the basal BG11 medium, NaNO_3_, Co(NO_3_)_2_, and ferric ammonium citrate are replaced by NaCl, CoCl_2_, and ferric citrate, respectively (21). Transcription was induced by washing and transferring the cells into a basal medium supplemented with 15.5 mM KNO_3_. All variants of the medium were buffered with TES, and there was little variation in pH (pH 7.60 in standard BG11; 7.73 in basal medium plus ammonia; and 7.77 in basal medium plus nitrate). Cells were harvested after 2-h incubation.

### mRNA-FISH

mRNA-FISH used the protocol of Skinner et al. (34) with some modifications (13). A set of 40–48 oligonucleotide probes (each probe 20 nucleotides with GC content ~50%) was designed against the target mRNA, with at least two bases separating probe sites. The probes were labeled with either TAMRA or FAM fluorophores at the 3′ end. The probe set was designed with the Stellaris RNA-FISH Probe Designer program (https://www.biosearchtech.com/stellaris-designer) and purchased from LGC Biosearch Technologies. Probe sets are listed in Table S2. The protocol for cell fixation, permeabilization, hybridization, and preparation for microscopy was as previously described (13).

### Confocal microscopy and image analysis

Imaging used a Leica TCS-SP5 laser-scanning confocal microscope with a 63× oil-immersion objective (numerical aperture 1.4). The confocal pinhole was set for ~0.72 µm section thickness in the *z*-direction. Images were recorded in 12-bit, 1,024 × 1,024-pixel format (with each pixel 24 × 24 nm) and acquired with 16× line averaging at 400 Hz line scan speed. Chlorophyll and phycocyanin fluorescence were detected using, respectively, 488 nm or 561 nm excitation, with emission detected at 660–720 nm. Detection of TAMRA and FAM-labeled FISH probes used, respectively, 561 nm excitation with 565–580 nm emission and 488 nm excitation with 510–540 nm emission. GFP detection was with 488 nm excitation and emission at 503–515 nm. Images were processed with the Fiji ImageJ package (41). To reduce noise, images were blurred (below optical resolution) over a 2 × 2-pixel window. For the FISH images, the autofluorescence background was subtracted out as in reference (13). For the *rbcL-gfp* strain, previously published sets of GFP fluorescence and *rbcL* FISH images (13) were re-analyzed.

mRNA colocalization analysis used the Fiji ImageJ plugin EzColocalization (42). Cell outlines were generated from a binary image obtained from the phycocyanin (PC) channel. The ImageJ Watershed function was used to separate cells in contact. The default threshold algorithm in EzColocalization was used to identify the cell areas. To calculate the metric matrices for two reporter channels, the Pearson correlation coefficient (17, 42, 43) metrics were used at Costes’ threshold (44).

To measure the position of mRNAs relative to the thylakoid membrane, a line 8 pixels wide was drawn across the short axis of the cell to generate a fluorescence profile showing two peaks from the thylakoids. To compensate for variable cell width, the distance between the thylakoid peaks was normalized. Interpolation in Microsoft Excel was used to set the same number of data points between the two thylakoid peaks for all the cells. The peak position of the mRNA FISH signal was then measured and plotted.

Mean cellular fluorescence intensity was measured after determining the cell area by thresholding the thylakoid (PC or Chl) channel. Cells not completely in the field of view were excluded from the measurement. To estimate the mean fluorescence intensity in the plasma membrane region, the thylakoid region was first selected from the Chl channel and then a band 0.2 µm wide was drawn around the thylakoid region using the Make Band function in ImageJ. Fluorescence in the band region was then quantified.

The normalized standard deviation of fluorescence images of cells was used as a measure of patchiness in the distribution of the fluorophore (for RplL-GFP in Fig. S2C and the low-abundance *nrtB* mRNA-FISH signal in Fig. 2B). Cell outlines were generated as described above. The standard deviation of each cell image was then computed with ImageJ and normalized by dividing by the total cell fluorescence.

For image presentation, all parallel samples are shown on the same color scale. Statistical significance was assessed from *P*-values obtained from two-tailed Student’s *t*-tests, carried out with Microsoft Excel software.

### Super-resolution microscopy

Images were acquired with an Elyra 7 microscope (Zeiss) with a Lattice Structured Illumination Microscopy (SIM^2^) module. Cell suspensions were spotted on a slide and covered with a 170 ± 5 µm high precision cover glass (Marienfeld). A Plan-apochromat 63×/1.4 DIC M27 objective lens was used. Chl and GFP were excited with a 488 nm laser with an exposure time of 100 ms. Fluorescence emission channels were separated with a 560 nm beam-splitter. Emission wavelengths were defined by a 495–550 nm bandpass filter for GFP and a 655 nm long-pass filter for Chl.

### Cell fractionation and Western blotting

Cells were collected during mid-exponential growth and broken by vortexing with glass beads (150–212 µm diameter) in isolation buffer (25 mM MES/NaOH pH 6.5, 10 mM CaCl_2_, 10 mM MgCl_2_, 25% glycerol, and protease inhibitor, Roche). Vortexing was done in five consecutive cycles at 4°C, each cycle consisting of 1 min of vortexing followed by 1 min of rest. Unbroken cells and glass beads were removed by centrifugation (400 *g*, 1 min). Membrane and soluble fractions were then separated by centrifugation at 16,000 *g* for 20 min at 4°C. Protein concentrations were measured with the Pierce BCA protein assay kit (Thermo Scientific). In total, 10 µg protein per sample was used for SDS-PAGE separation. GFP was detected by western blotting using an Anti-GFP primary antibody (Abcam, 1:5,000 dilution in 10× PBS, 0.8% Tween 20, and 5% milk) followed by the IRDye 800CW Goat anti-Rabbit IgG secondary antibody (LI-COR, 1:5,000 dilution in 10× PBS, 0.8% Tween 20 and 5% milk).

## SUPPLEMENTAL MATERIAL

Supplemental figures and tables jb.00209-23-s0001.pdf Figures S1 to S3, Tables S1 and S2.

ASM does not own the copyrights to Supplemental Material that may be linked to, or accessed through, an article. The authors have granted ASM a non-exclusive, world-wide license to publish the Supplemental Material files. Please contact the corresponding author directly for reuse.
