# Supplementary material for: Locations of membrane protein production in a cyanobacterium
Source: J Bacteriol. 2023 Oct 3;205(10):e00209-23. doi: 10.1128/jb.00209-23 (PMC10601611; doi:10.1128/jb.00209-23)
Supplement: Supplemental figures and tables — Figures S1 to S3, Tables S1 and S2. [file jb.00209-23-s0001.pdf]

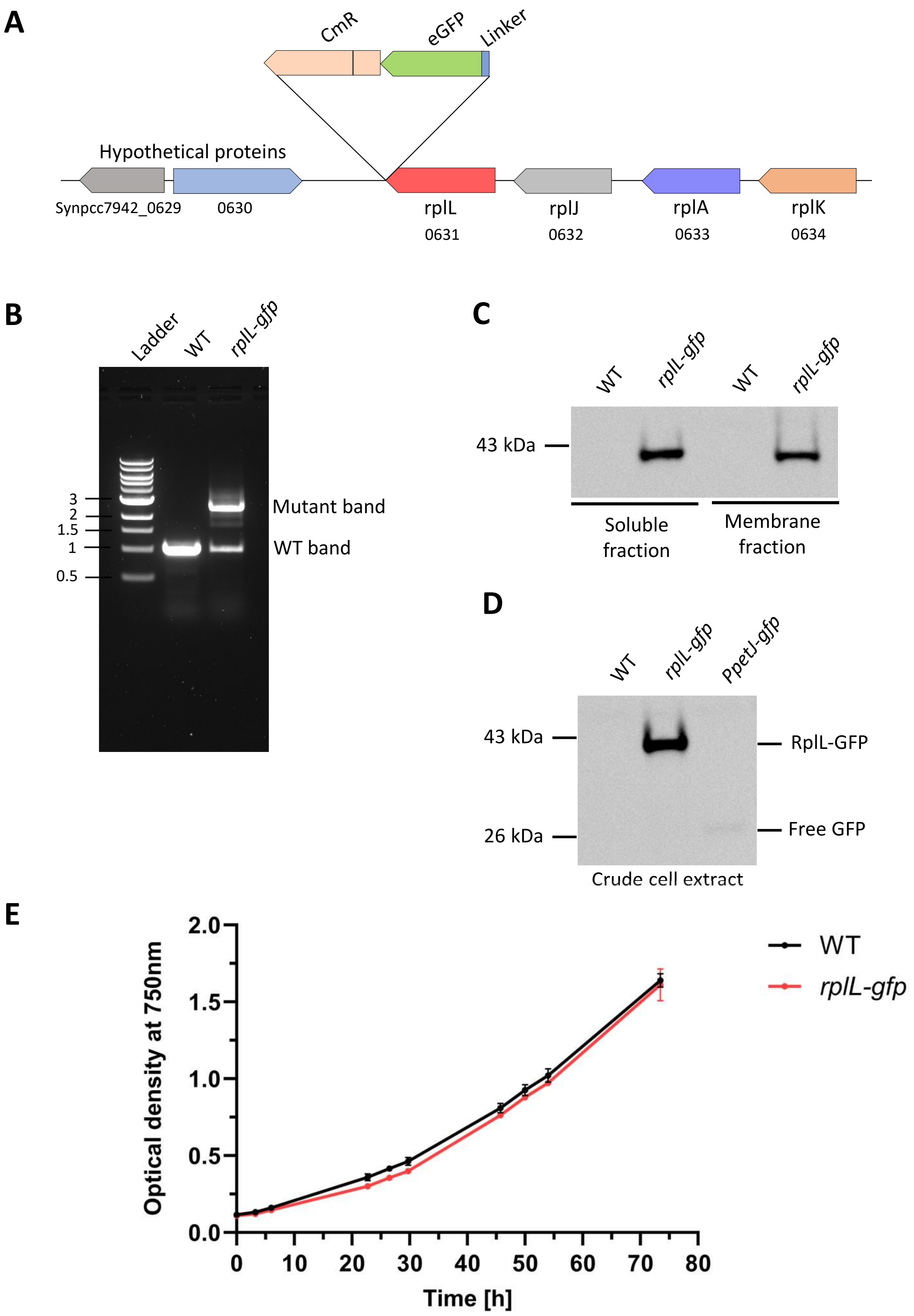

FIG S1 Characterization of the *Synechococcus rplL-gfp* mutant (A) Map of the genomic region. **(B)** Segregation status of the mutant probed by PCR (ladder fragment sizes in kb). **(C)** Western blot with anti-GFP antibody showing the presence of RplL-GFP in the mutant in both the soluble and membrane fractions (total protein 10 µg per lane). **(D)** Western blot showing absence of free GFP in the crude cell extract. Cells from the wild type (WT) and a strain expressing free GFP driven by PpetJ promoter (*PpetJ-gfp*) are used as controls. **(E)** Growth curve for *rplL-gfp* vs WT under standard conditions as described in Materials and Methods. Error bars indicate standard deviations from 3 biological replicates.

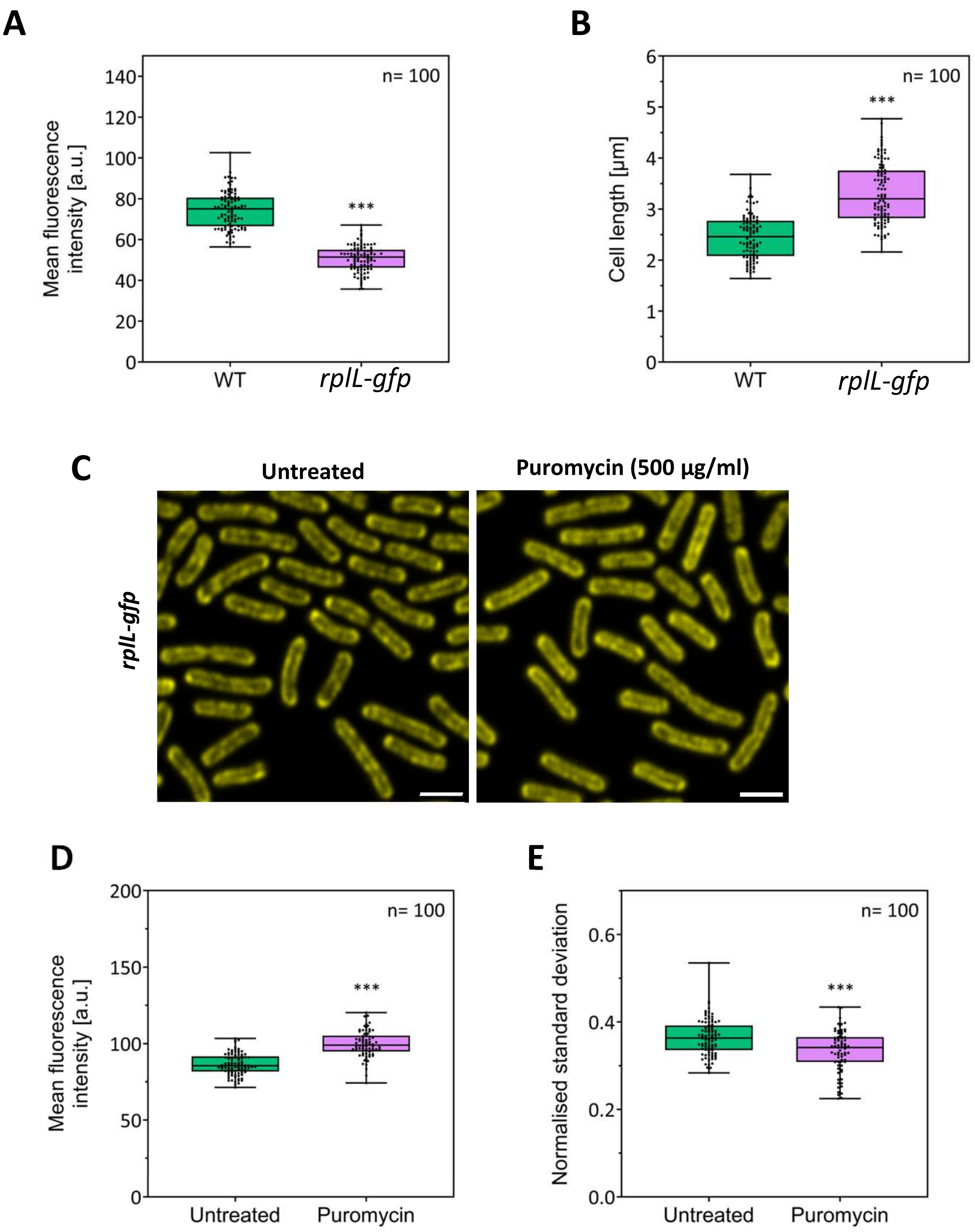

FIG S2 Phenotype of the *Synechococcus rplL-gfp* mutant (A) Mean cellular chlorophyll fluorescence intensity ($p = 5 \times 10^{-51}$ for wild type (WT) vs. *rplL-gfp*) **(B)** Mean cell length ($p = 1 \times 10^{-23}$ for wild type vs. *rplL-gfp*) **(C)** Fluorescence micrographs showing the GFP signal in *rplL-gfp* cells untreated or exposed to puromycin (500 µg ml^{-1} for 1 hr). Scale-bars 2 µm. **(D)** Quantitation of the cellular GFP fluorescence in *rplL-gfp* cells untreated or exposed to puromycin ($p = 2 \times 10^{-19}$). **(E)** Effect of puromycin treatment on the sub-cellular distribution of RplL-GFP fluorescence, with patchiness of the signal quantified from the normalized standard deviation of the fluorescence image. $p = 1.5 \times 10^{-5}$. Error bars in the box plots indicate the range of values recorded, the center line shows the median and the box spans the interquartile range, n: the number of cells measured, ***= significant difference, at $p < 0.001$, measured by unpaired two-tailed Student's t-test.

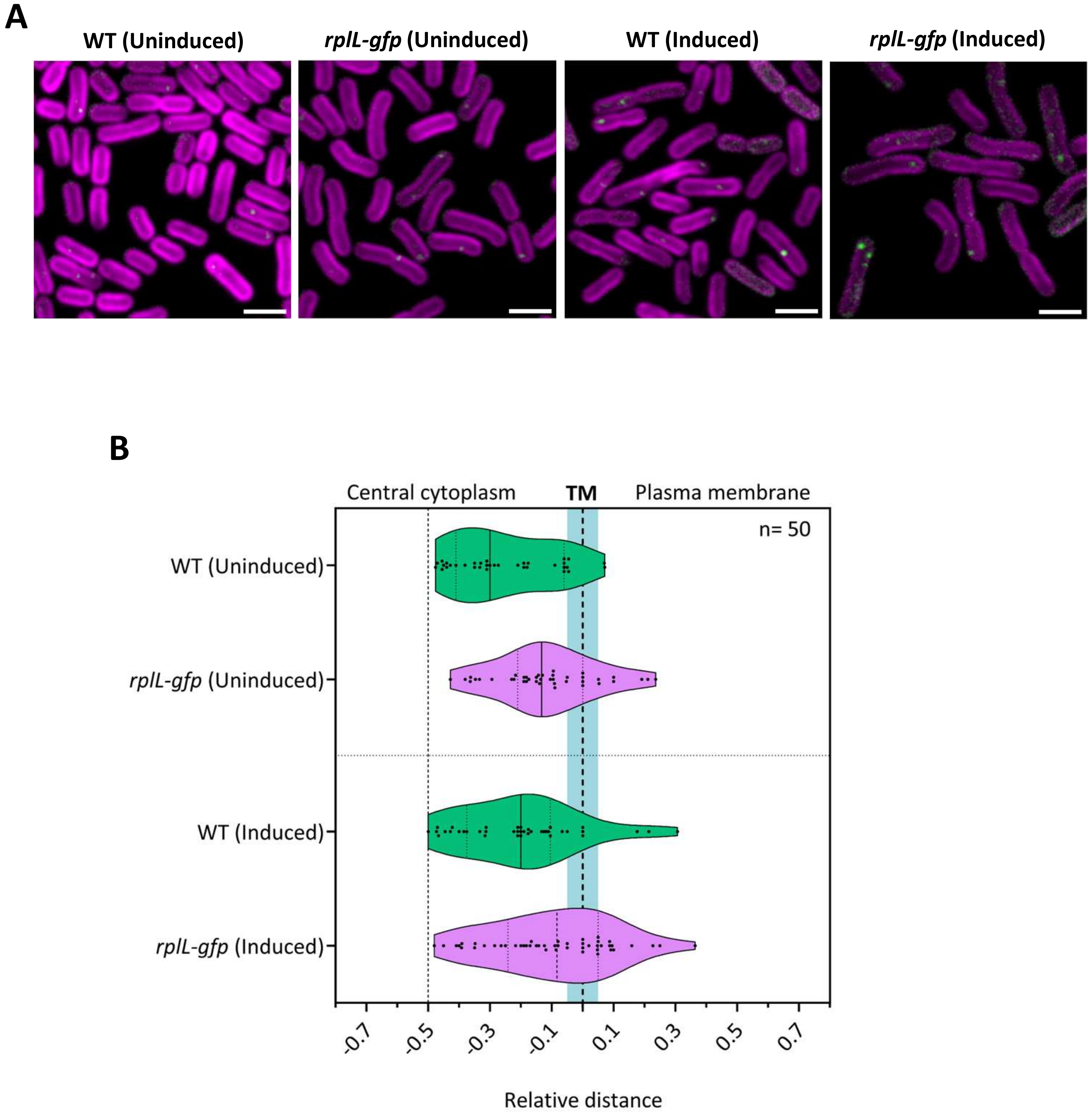

FIG S3. Location of *nrtB* mRNA relative to the thylakoid membrane in *Synechococcus rplL-gfp* cells. **(A)** Fluorescence micrographs showing FISH signals from *nrtB* mRNA (in green) relative to thylakoid membrane autofluorescence (TM, magenta), comparing *rplL-gfp* with wild type (WT). Cells were uninduced or induced for *nrtB* expression as in Figs 2 and 3. Scale-bars 2 µm. **(B)** Violin plots showing the radial distance of FISH foci from the center of the cell, relative to the thylakoid membrane peak.. n = number of cells measured.

.

Table S1: Oligonucleotide primers used to generate the *rplL-gfp* strain

**Purpose**		**Sequence (5' to 3')**
Amplification of 500bp sequence downstream of *rplL* (SynPCC7942_0631)	Forward primer:	AGGGCGGGGCGTAATTCGAGTAGCTTGAAGGCAGGAC
	Reverse primer:	AGAATACTCAAGCTCCGGTCACCGCCGGTAGC
Amplification of the end 500 bp of *rplL*	Forward primer:	AGGGCGAATTGTCCCGAATTCTTTGGCAC
	Reverse primer:	TCACAGGTAGTTCAGGACCAGGTAGCTTGACGCTGACTTTGGC
Amplification of eGFP	Forward primer:	CTACCTGGTCCTGAACTACCTGTGAGCAAGGGCGAGGAG
	Reverse primer:	TGCCGATCACTCGAGCTACTTGTACAGCTCGTCCATG
Amplification of CmR	Forward primer:	TGTACAAGTAGCTCGAGTGATCGGCACGTAAGAGGTTC
	Reverse primer:	TTCAAGCTACTCGAATTACGCCCCGCCCTGCCACTC
Amplification of pGEM-T Easy	Forward primer:	CGGTGACCGGAGCTTGAGTATTCTATAGTGTCACC
	Reverse primer:	TGCCAAAGAATTCGGGACAATTCGCCCTATAGTGAGTC

Table S2: mRNA-FISH probe sets

A. Oligonucleotide probes designed against the *Synechococcus psbAI* (Synpcc7942_0424) gene sequence.

Probe number	Sequence (5' to 3')	Probe number	Sequence (5' to 3')
1	TCGCGAAGAATGCTGGTCAT	21	ACATGAAGTTGAAGGTGCCG
2	GATCCCAAACGTTATCGCGG	22	TTGTGCTCTGCTTGGAACAC
3	CTGGTTACCCACTCACAAAA	23	TGTGGAAGGGGTGCATCAAA
4	CGAACCAACCCACGTAGATG	24	AGAACAGCGAACCACCGAAC
5	AGAGTGGGGATCATCAGCAC	25	CACCAACGAACCGTGCATTG
6	ATGAAGCAGATGGTGGCGGT	26	TAGTTTTGGCTCTCGGTCTC
7	AGGGGCTGCAATGAACGCAA	27	CTCTTGACCAAATTTGTAGC
8	ATACATGAGAGAGCCGGCAA	28	CCACGATGTTGTAGGTCTCT
9	CGCCGGAAATGATGTTGTTG	29	ATCAAGCGACCGAAGTAACC
10	GCGTTGCTGGAAGGAACAAC	30	GTTGAACGATGCGTATTGGA
11	GCTTCCCAAATCGGATAGAA	31	GGAAGAAGTGCAGCGAACGG
12	TTGTACAGCCACTCGTCGAG	32	CATGGAGGTAAACCAGATGC
13	CCACTAATTGGTAAGGACCA	33	TGAACGCCATGGTGCTGATG
14	ATACCCAGCAAGAAGTGGAA	34	ACTGGTTGAAGTTGAAACCA
15	ATTGACGACCCATGTAGCAG	35	TTGCCTTGGCTATCCAAAAC
16	ATACCGAGGCGGTACGACAG	36	ATCTGCCCAAGTGTTGATCA
17	ATGCAACACAGATCCAAGGG	37	CCAAGTTGGCACGGTTCAAC
18	AAAAGCAGCCGAGAGTGGAG	38	ATTACGCTCGTGCATCACTT
19	CCGATCGGGTAGATCAGAAA	39	AAGTCGAGCGGGAAGTTGTG
20	CATGCCGTCCGAGAACGAAC	40	ATTGAAGGCGCAGTCAAAGC

Table S2: mRNA-FISH probe sets

B. Oligonucleotide probes designed against the *Synechococcus psaA* (Synpcc7942_2049) gene sequence.

Probe number	Sequence (5' to 3')	Probe number	Sequence (5' to 3')
1	GTCGGAACCGGATTTTTATC	25	CTAGGTTGATCGACAGTTGG
2	CTTACCCCACTTCTCAAAAG	26	ACGATGATGCTGATCGAACC
3	TCCAAATCCAAGTTGTGGTT	27	AAGTACGGATACGGAGGCAT
4	AAATCGTGAGCGTTAGCGTG	28	CAATCCAGATGTGGTGAGTA
5	TCAAGGTCACTGGTATGACT	29	TGAAAATGGCAGCGTGAGCA
6	ACCAAAGTGAGCGCTGAAGA	30	AGGAGGTTGTCGACATTCTT
7	ACCAGATAAAGATCACCGCA	31	GTGTCGTTGTGGATGTAGAG
8	CAGCCGCTGAAATTGGAGAA	32	CGAGAACATGTCTTGGGGAC
9	GAAGATCGGCCAAACGACTT	33	AAGATGGGCTGCAGTTGAAT
10	GATCTGAATGCCATGGAAGC	34	GTGCAAGGGCATGAATGTTT
11	CCGTGACGTAGAGCTGAAAC	35	CAAACACTTGGCTGACCGAA
12	CTTTGTGGTAGTGGAACCAG	36	GAATCGTGAAGGCGTGGATG
13	TCGACGTTTTGGAACCATTC	37	GAACTCCGAGCGTAGAGAAC
14	CAAGTGGTGGTTCAACATCG	38	AAGGTTGGCTTTGTCAGGAA
15	CGACACGTGAATCTGGTGAC	39	GAACAAGCCTAGGAACACGT
16	TTTTGCCGTTGAGAACCAAC	40	TCGACAGGGAGTTGTACATC
17	CGGAATATCAGCCGCAGAAG	41	CCAAACATCGGACTGCATTT
18	TCAGGCTGACATCCAAGAAC	42	TTGAGCAAAGTTGCCATTCG
19	AGCGTGAAGAAGGCTTTCAC	43	GACAAAGTGAGCACCCAAGA
20	ACCTTTGAAGGTCAGGAAGT	44	GCCACTGAACAGGAACATCA
21	GTGACCCGCAACAATGAAGA	45	CAGACGATGGACTCGATCAG
22	CCAAGATTTCTTTGAGGCTG	46	CACTTTGAGCTTGTTGTGAG
23	GTGAAAGGACCTTTGTGAGC	47	AGACCATGTGGTCACAATTC
24	TCTCATAGAGACCTTTGTGG	48	AACCAACTGCAATGATGCGG

Table S2: mRNA-FISH probe sets

C. Oligonucleotide probes designed against the *Synechococcus psbDC* (Synpcc7942_0655-56) gene sequence.

Probe number	Sequence (5' to 3')	Probe number	Sequence (5' to 3')
1	AGTCGTCGAGGACGTCAAAC	25	AAGACGATCAAGCCAGCATG
2	CTGACCAACCCACAAATACA	26	TCGGTTTTTCGGGGACAAAG
3	CCAGTGCTAAATACGCACAG	27	AAGTGCGAGAGCAGGATGAT
4	GTGTACCACGACGTCACAAA	28	GGAAGGTATCGACGACTTCG
5	AAAAGTTGCCGCCTTCTAAG	29	AGAAATGAGGTGCAGAACCC
6	ACAGCAGCATCAACGAATGC	30	AGTCTTGGCTGAAGAAGGTG
7	AGTGCTACGAAGTTCCACAA	31	ACCAATGATGTTGGTCATCT
8	GCGCAATCTCAAATTGACGC	32	GAACATGGCCTTAAAGACCA
9	GAAAAGGCGATCGCGTTGTA	33	TTGGAGATGATGCGGACATC
10	GGGTACATCAAGAACACCGA	34	AAAGGGTGATTTCAGCAGGT
11	AAAGCTCGGAGCGAAGAACC	35	ATCACGTCTTCAAGGTTGTC
12	ACCCTTGCAGGAACAACAAA	36	ACCCGAAATGCAGATCAGAC
13	CATCATGTGGAATGGGTTCA	37	AAAGGCTTGGTCAGGATGTG
14	GATTGCTCTGAATCCTCGAA	38	CTGTAGGAGAGGTAAGCTTC
15	ATCTGGCTCCAAAAACGGTT	39	ATGAAGCCCATCAACGACAG
16	TGTTCGAAAACGCAATCCCG	40	GACGGTGTTGTTGTACCAAA
17	ATGAAAAAGTGCAGCCACCG	41	GGCCAAAGAACTCGGAAGGA
18	GTTCAACGCCAAACCTACAA	42	TCACGCACCAAGAAGGTGAA
19	TCTTCGTGTAGAACGTCTCA	43	CTTGAGCTGAACCGATGTTG
20	ATCCCTTCGTTCAACAAGAT	44	AGAGCGCATCAGGTATTTAC
21	ACGAATTTTTCGTGCGGTTG	45	AATGTCATTGGTCAGCTTGT
22	TGCGATCACGGAAGGACTAG	46	ACACGAAGTTCACCGAGTTG
23	GTAACCGGTGGAGTCAATAT	47	AGAAGGCCAAGACGAAGTGG
24	TTACCGGACAGGTTGATCAA	48	GATACCTTTCTCAAAGCCTG

Table S2: mRNA-FISH probe sets

D. Oligonucleotide probes designed against the *Synechococcus nrtB* (Synpcc7942_1237-38) gene sequence.

PROBE NUMBER	SEQUENCE (5' TO 3')	PROBE NUMBER	SEQUENCE (5' TO 3')
1	CAGAGCGACGGACAGAACTG	25	GCATCCCAAATAAAGTAGCC
2	GAGCTTTGGATTTTTAACCC	26	AGAATAATCTGGCTCGAGCT
3	ACAACGTAGGGCAGAAAGGG	27	CCGACTAAACCGACGTAGAA
4	TCGCCAGAAAAATTGGCAGG	28	CACGAGGCGATCGAGACTAA
5	AGGATGGCCGAGATCACTTG	29	CGACTGACAGGACTGACGAG
6	TTGGCCACAACGTTGATCGG	30	CAGACATGGCGATAGGGCTC
7	GACGATGTAGGGCATCCAAG	31	ACGTGGTCAACCGCTAAGAA
8	GTACCGCCGTTATCAAAGAA	32	CGGTAGGTCAAAGACCTGAT
9	CAAAATCTGGAGGCCGAGAC	33	TTGAGGGCGATATATTGTCC
10	AAGAGATAGCCGATCGCTAC	34	CCGGGGCGAATATTCAAACT
11	TAGAACACCACCGACCAAGA	35	AGTGGCCAATCAAGGAGATA
12	TTGCCCAGGAATTTACTCAT	36	CAGCGTCGATTTACCACAGC
13	TGTTCGCAAGACTTGGATCA	37	CAAGGCCAGCAATCAAGTTG
14	GAGATAGGGAACCAAGCCAA	38	GACCTTCAAGAATGATGCCG
15	GGCATCTTGAAACACCATCA	39	TGGAAAACCACCATGCGATC
16	GATGACGAAGATTGCCGAGG	40	CCACGGTAAGAGCGAATAGT
17	GATAATGATCGGCCAGATCG	41	CGATATTCTGGCGGACAGTT
18	GGGAATTTGGTTAATTCCCA	42	ATTGCGATCGTGCAGAACAC
19	GCGAGCAACGTTGTTATAGT	43	TTTCTTCGATAATCGTGCGG
20	CTTTTTTGCTCAGCTTTAGG	44	CCGCAACCCAACAAGATCAA
21	ATCAGGATGTTGAGGATGTA	45	CGGAAATCTCGTGGGGATAT
22	AACGTAGGGAACGGTCGAAG	46	TCTAGGAGTAGCAGTTTGGG
23	CAACCGCAATTCTCAAACCT	47	ATCCAAGGCACCAAAGGGTT
24	TTTGAGCATCTCAGCAGCGA	48	ATCAGTTGCTCTTGCAGGTT
